# LON is the master protease that protects against protein aggregation in human mitochondria through direct degradation of misfolded proteins

**DOI:** 10.1038/srep17397

**Published:** 2015-12-02

**Authors:** Ayenachew Bezawork-Geleta, Erica J. Brodie, David A. Dougan, Kaye N. Truscott

**Affiliations:** 1 Department of Biochemistry and Genetics, La Trobe Institute for Molecular Science, La Trobe University, Melbourne 3086, Australia

## Abstract

Maintenance of mitochondrial protein homeostasis is critical for proper cellular function. Under normal conditions resident molecular chaperones and proteases maintain protein homeostasis within the organelle. Under conditions of stress however, misfolded proteins accumulate leading to the activation of the mitochondrial unfolded protein response (UPR^mt^). While molecular chaperone assisted refolding of proteins in mammalian mitochondria has been well documented, the contribution of AAA+ proteases to the maintenance of protein homeostasis in this organelle remains unclear. To address this gap in knowledge we examined the contribution of human mitochondrial matrix proteases, LONM and CLPXP, to the turnover of OTC-∆, a folding incompetent mutant of ornithine transcarbamylase, known to activate UPR^mt^. Contrary to a model whereby CLPXP is believed to degrade misfolded proteins, we found that LONM, and not CLPXP is responsible for the turnover of OTC-∆ in human mitochondria. To analyse the conformational state of proteins that are recognised by LONM, we examined the turnover of unfolded and aggregated forms of malate dehydrogenase (MDH) and OTC. This analysis revealed that LONM specifically recognises and degrades unfolded, but not aggregated proteins. Since LONM is not upregulated by UPR^mt^, this pathway may preferentially act to promote chaperone mediated refolding of proteins.

To be functionally active most proteins must fold into distinct three-dimensional structures. However, within the crowded environment of the cell, proteins are highly susceptible to intermolecular aggregation, not only during *de novo* folding but also as a consequence of unfolding caused by a destabilising stress^1^,^2^,^3^,^4^,^5^. Protein aggregation results in a loss of protein function and if left unchecked can disturb cellular processes leading to cytotoxicity. To prevent the accumulation of these toxic protein aggregates within the cell, organisms have co-evolved complex protein quality control (PQC) networks composed of molecular chaperones and proteases^2^,^3^,^6^. Molecular chaperones are not only responsible for the *de novo* folding of proteins but also for their refolding after stress. Proteolytic machines on the other hand, are responsible for the removal of damaged or unwanted proteins. In general, these proteolytic machines belong to the AAA+ (ATPases associated with a variety of cellular activities) superfamily and as such are referred to as AAA+ proteases^7^,^8^,^9^. Some of these ATP-dependent machines contribute to the clearance of damaged cellular proteins by degrading them into short peptides and thereby preventing the detrimental accumulation of protein aggregates. To coordinate the cell’s defence against the accumulation of damaged proteins, organisms have also evolved a number of integrated stress response pathways such as the heat shock response, envelope stress response and unfolded protein response in the ER^3^,^10^,^11^,^12^. These stress response pathways mediate the up-regulation of molecular chaperones and proteases and in some cases attenuate translation in an attempt to re-establish protein homeostasis. In many cases PQC components also play an integral role in the sensing and signalling of the stress^2^,^3^,^10^,^11^,^12^.

In mammalian mitochondria the PQC network contains key chaperones such as mtHSP70 (also known as HSPA9 and mortalin) and HSP60 (also known as HSPD1) and their cofactors and five different AAA+ proteases. Two of these proteases, LONM (also known as LONP1) and CLPXP, are located in the matrix while intermembrane space facing YME1L1 (also known as *i*-AAA protease) and two different matrix facing *m*-AAA protease complexes are anchored to the inner membrane^13^,^14^. Consistent with an important role in maintaining mitochondrial protein homeostasis, inherited mutations in PQC components are associated with several diseases^15^,^16^,^17^,^18^,^19^,^20^. Apart from naturally occurring mutations, a disruption to mitochondrial protein homeostasis is achieved experimentally by altering the stoichiometry of nuclear and mitochondrial encoded protein subunits of various respiratory chain complexes^21^,^22^,^23^,^24^ or by overexpressing a folding-defective protein within the mitochondrion^25^. In higher eukaryotes, this disturbance to protein homeostasis within the mitochondrion is sensed, and a mitochondria-to-nucleus signalling pathway is activated. This pathway, known as the mitochondrial unfolded protein response (UPR^mt^), was initially identified in a monkey cell line, by over-expression of a folding-incompetent form of the mitochondrial protein, ornithine transcarbamylase (OTC) termed OTC-Δ^25^. This pathway has subsequently been described in human and mouse cells lines as well as in *Drosophila melanagaster* and *Caenorhabditis elegans*^24^,^26^,^27^,^28^. Although the UPR^mt^ signalling pathway is only poorly understood in mammals, the pathway and many of its components have been defined in *C. elegans*^29^,^30^,^31^,^32^. Recent data suggests that the subcellular distribution of ATFS-1 (activating transcription factor associated with stress-1) is a key regulatory branch-point in the pathway^32^. Under non-stress conditions ATFS-1 is directed to the mitochondrial matrix by an N-terminal mitochondrial targeting signal, where it is rapidly degraded by the AAA+ protease, Lon. Under conditions of stress however, ATFS-1 import (into mitochondria) is inhibited and it is instead directed to the nucleus by a nuclear localisation signal, resulting in the up-regulation of nearly 400 genes^32^. Significantly, the redistribution of ATFS-1 to the nucleus is dependent on the inner membrane embedded ABC-family peptide transporter Haf-1 (Haf transporter 1). Furthermore, both ClpX and ClpP from *C. elegans* have been experimentally implicated as direct components of the UPR^mt^ signal transduction pathway^30^,^31^. Interestingly, Haynes and colleagues proposed a model for *C. elegans*, whereby stress within the mitochondrial matrix, is signalled across the inner membrane (via Haf-1) by a factor that is generated by the ClpXP protease^31^. Hence a ClpXP-dependent degradation product may regulate ATFS-1 trafficking.

There is growing evidence that the mitochondrial unfolded protein response is associated with several pathophysiology conditions such as inflammatory disease, infection, cancer and neurodegeneration^33^,^34^. It is currently a focal point in mitochondrial theory of aging and has potential to be manipulated as a strategy to reduce age-dependent cellular decline. Despite this, and the detailed understanding of this signalling pathway in worms, our understanding of the pathway in mammals remains rudimentary. In mammals the pathway is triggered by overexpression of OTC-Δ, which engages with mitochondrial PQC components such as HSP60^25^. The transient expression of OTC-Δ in mammalian cells leads to the selective transcriptional up-regulation of nuclear genes encoding mitochondrial chaperones (HSP60, HSP10 and mtDnaJ) and proteases (CLPP, YME1L1 and MPPβ) as well as the nuclear transcription factor C/EBP homology protein (CHOP/DDIT3)^25^,^35^, while the expression of other prominent PQC proteins such as mtHSP70 and LONM are not effected. The mechanisms of sensing the increased load of unfolded protein and transfer of the signal across the two membranes of the mitochondrion to the cytoplasm however is not yet understood. Nevertheless, given that human mitochondrial CLPXP is known to recognise and degrade the model unfolded protein, casein and mammalian CLPP is transcriptionally upregulated upon over-expression of OTC-Δ^25^,^36^,^37^, it is reasonable to suggest that human CLPXP may play an important role in the degradation of misfolded proteins and subsequent stress signalling as reported in *C. elegans*.

In addition to CLPXP, *C. elegans* and mammalian mitochondria both harbour soluble Lon family AAA+ proteases. Mammalian mitochondrial LONM degrades proteins that are mildly damaged by oxidation, unassembled native proteins, as well as natively folded proteins for regulatory purposes^38^,^39^,^40^,^41^,^42^,^43^. However, it is not known if LONM and CLPXP both act to clear unfolded or aggregated proteins from the mitochondrial matrix. Here we report on a systematic investigation of the contribution of LONM and CLPXP to the processing of UPR^mt^-inducing protein OTC-∆ and the nature of the conformational state of the proteins that are recognised. Our data suggest that LONM, and not CLPXP, is primarily responsible for degradation of misfolded protein in the mitochondria matrix. Thus UPR^mt^ stress signalling by the direct and promiscuous degradation of unfolded proteins by CLPXP may not be a conserved mechanism.

## Results

### OTC-Δ is rapidly degraded in mitochondria in an ATP-dependent process

**Figure 1:**
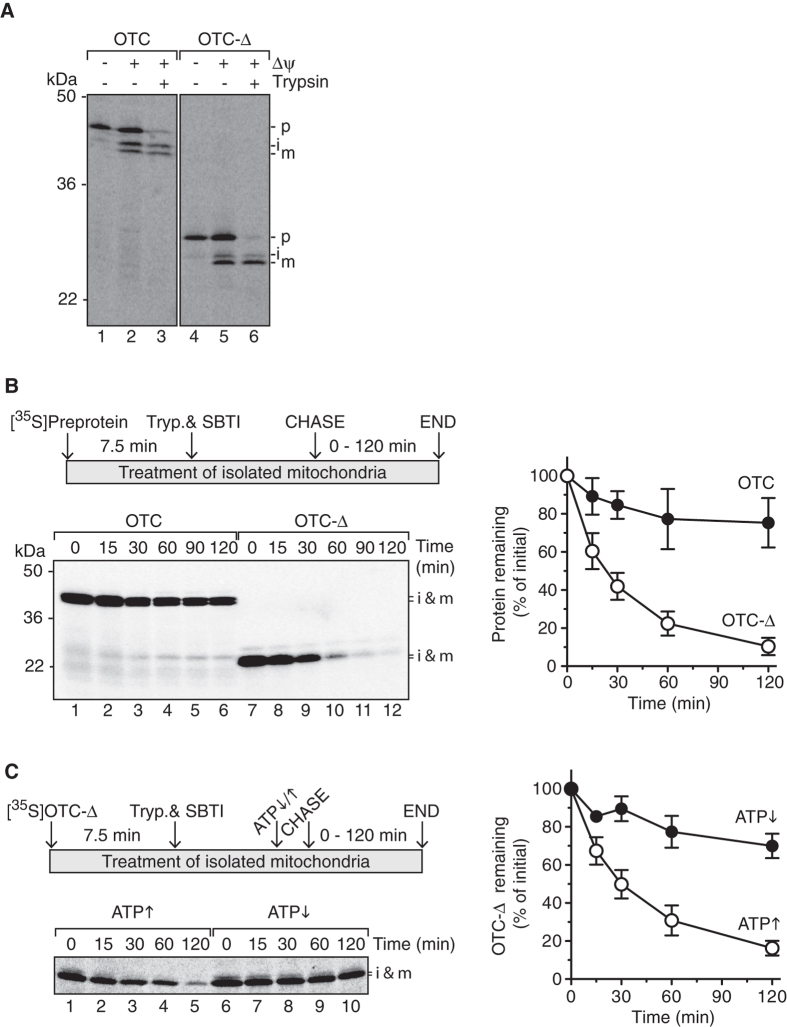
OTC-∆ is rapidly degraded in mitochondria in an ATP-dependent manner. (**A**) Import of [^35^S]OTC and [^35^S]OTC-∆ preproteins into isolated mitochondria (7.5 min) with and without membrane potential and trypsin treatment as indicated. Import was analysed by digital autoradiography following separation by SDS-PAGE. (**B**) Turnover of [^35^S]OTC-∆ (lanes 7–12) relative to [^35^S]OTC (lanes 1–6) in mitochondria analysed by import-chase assay (top panel). The bottom left panel shows a representative digitial autoradiogram of the assay following separation by SDS-PAGE while the right panel is a quantification of four independent experiments. (**C**) Turnover of [^35^S]OTC-∆ in mitochondria analysed by import-chase assay with high (↑) or low (↓) ATP levels. Samples were analysed by digital autoradiography following separation by SDS-PAGE (representative strip is shown in bottom left panel) and quantitation of three independent experiments (right panel). Error bars shown in Fig. 1 represent standard error of the mean (SEM). p, preprotein; i, processing intermediate; m, mature protein.

To study the stability of OTC-∆, relative to wild-type OTC, we initially employed an import-chase proteolysis assay^38^,^44^. For this purpose, radiolabeled preprotein (either OTC or OTC-Δ) was incubated with mitochondria isolated from HeLa cells. As expected, OTC preprotein was processed into an intermediate (~38 kDa) and a mature (37 kDa) form of the protein^45^ when incubated with energised mitochondria (Fig. 1A, lanes 2 and 3). Importantly, these data also demonstrated that radiolabeled OTC-Δ (synthesised in rabbit reticulocyte lysate) was not only competent for *in vitro* import into mitochondria, but also correctly processed into the intermediate and mature forms of the protein (Fig. 1A, lanes 5 and 6).

Next, to assess the stability of OTC-Δ relative to OTC, an import-chase proteolysis assay was performed as outlined (Fig. 1B, top left panel). This analysis revealed that OTC-∆ is highly unstable in isolated mitochondria (Fig. 1B, bottom left panel) with only ~10% of the protein remaining after 120 min (Fig. 1B, right panel, open circles) compared to ~80% remaining for OTC at the equivalent time (Fig. 1B, right panel, filled circles). This *in vitro* analysis, which permits an assessment of protein turnover independent of other cellular processes such as mitophagy, revealed that OTC-∆ was degraded, within mitochondria, with a half-life of ~25 min (compared to a half-life >120 min, for wild type OTC, under the same conditions).

To determine if the turnover of OTC-Δ was mediated by a mitochondrial ATP-dependent protease, we performed an import-chase proteolysis assay following the manipulation of ATP levels within mitochondria (Fig. 1C, left panels)^38^,^46^. In the presence of “*high*” levels of ATP, OTC-Δ was rapidly degraded with less than 20% remaining after the 2 h chase (Fig. 1C, right panel, open circles). In comparison, depletion of ATP from mitochondria strongly inhibited the turnover of OTC-Δ, with ~70% of the protein remaining after the 2 h chase (Fig. 1C, right panel, filled circles). Collectively, these data demonstrated that OTC-Δ is rapidly degraded in mitochondria and that its turnover is mediated by an ATP-dependent protease.

### Depletion of mitochondrial matrix AAA+ proteases reveals a lack of functional overlap in the clearance of OTC-Δ

**Figure 2:**
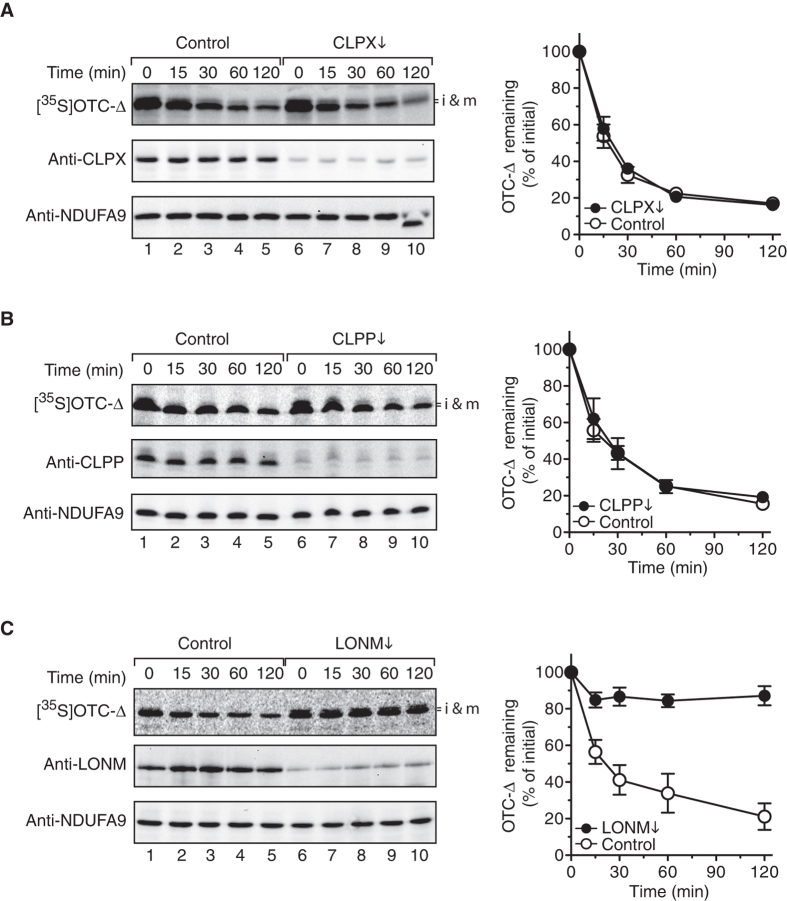
The AAA+ protease LONM mediates turnover of OTC-∆ in mitochondria. Representative strips from import-chase assays (left panels) showing turnover of [^35^S]OTC-∆ imported into mitochondria isolated from HeLa cells transfected with negative control siRNA (Control) or siRNA against *CLPX* (CLPX↓, (**A**)), *CLPP* (CLPP↓, (**B**)) and *LONP1* (LONM↓, (**C**)). Samples were analysed by digitial autogradiography (top left panels) and immunoblotting (middle and bottom panels) of PVDF membranes following separation by SDS-PAGE and Western transfer. The quantification of three (**A,B**) or four (**C**) independent experiments for each experimental condition are shown in the right panels. Error bars represent SEM. i & m, intermediate and mature protein; NDUFA9, NADH dehydrogenase (ubiquinone) 1 α subcomplex 9, mitochondrial.

Mammalian mitochondria contain the two soluble matrix AAA+ proteases; CLPXP and LONM. The CLPXP protease consists of the peptidase CLPP, which is composed of two heptameric rings stacked back-to-back, and a hexameric unfoldase – CLPX, which can associate at either or both ends of CLPP^36^,^47^. The central component (CLPP) is essential for hydrolysis of peptide bonds, while CLPX uses the energy stored in ATP to drive the unfolding and translocation of proteins into the proteolytic chamber of CLPP^48^,^49^. To experimentally determine the relative contribution of CLPXP and LONM to the turnover of OTC-Δ, each component was depleted from mitochondria using RNA interference (RNAi) and an import-chase proteolysis assay of OTC-Δ performed (Fig. 2). Initially, the stability of OTC-Δ was monitored in mitochondria depleted of CLPX. Following import of OTC-Δ into CLPX-depleted mitochondria (CLPX↓), an import-chase assay was performed. Despite the successful knock down of CLPX in mitochondria (Fig. 2A, middle panel) the half-life of OTC-Δ didn’t change (Fig. 2A). Since CLPP, and not CLPX, is transcriptionally upregulated upon activation of UPR^mt^ we examined the possibility that CLPP could act independently of CLPX in the degradation of OTC-∆. Despite the successful knockdown of CLPP by RNAi- (Fig. 2B, middle panel) the rate and extent of OTC-Δ degradation in CLPP-depleted (CLPP↓) mitochondria (Fig. 2B, filled circles) was unchanged compared to control mitochondria (Fig. 2B, open circles). Collectively these data indicate that OTC-Δ is not a substrate of the mammalian CLPXP protease.

Next, to determine if the matrix AAA+ protease LONM was responsible for the turnover of OTC-∆, it was depleted from HeLa mitochondria using RNAi. The steady state level of mitochondrial LONM was highly reduced in the siRNA treated cells (Fig. 2C middle panel, compare lanes 6–10 with lanes 1–5). Importantly, the turnover of OTC-Δ was almost completely inhibited in LONM depleted-mitochondria (Fig. 2C top panel, lanes 6–10). Indeed, quantification of four independent import-chase proteolysis experiments, demonstrated that after the 2 h chase ~85% of the imported protein remained in LONM-depleted mitochondria (Fig. 2C, filled circles), while only ~20% of the imported protein remained in control mitochondria (Fig. 2C, open circles). Consistently, the half-life of OTC-Δ increased dramatically, from ~25 min in control mitochondria to >120 min in LONM depleted mitochondria. Collectively these data suggest that LONM, and not CLPXP, is responsible for the turnover of OTC-Δ in mammalian mitochondria.

### The LONM protease is responsible for degradation of OTC-Δ in mitochondria

**Figure 3:**
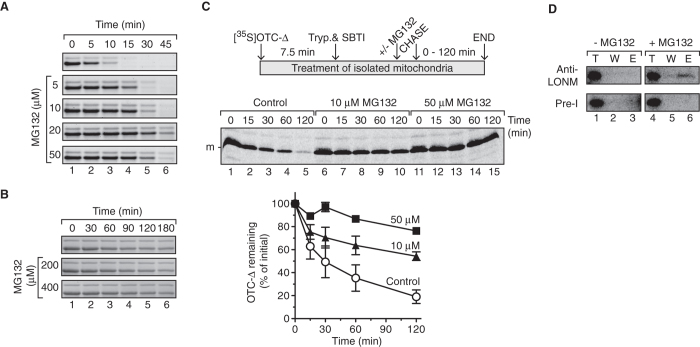
MG132 treatment of isolated mitochondria inhibits the turnover of imported OTC-∆ and traps it in complex with LONM. (**A**) *In vitro* degradation of α-casein by LONM in the absence and presence of MG132 as indicated. Relevant strips from Coomassie Brilliant Blue stained Tris-Tricine SDS-polyacrylamide gels are shown. (**B**) *In vitro* degradation of α-casein by CLPXP in the absence and presence of MG132 as indicated. Relevant strips from Coomaasie Brilliant Blue stained glycine SDS-polyacrylamide gels are shown. (**C**) Turnover of [^35^S]OTC-∆ in MG132 treated mitochondria monitored by import-chase assay. Samples were analysed by digital autoradiography (middel panel) following separation by SDS-PAGE and quantitation of three independent experiments (bottom panel). Error bars represent SEM. m, mature protein. (**D**) Immunoprecipitation of LONM analysing co-immunoprecipitation of [^35^S]OTC-∆ imported into isolated mitochondria in the presence and absence of MG132 as indicated. Pre-I refers to control immunoprecipitation using the pre-immune serum. Shown is the relevant section of the digital autoradiogram following separation by SDS-PAGE. T, 10% of total fraction; W, 90% of wash fraction; E, 90% of eluted fraction.

To exclude possible off-pathway effects associated with using RNAi, we sought to validate the import-chase proteolysis data using an alternative approach. To do so, we used MG132, an inhibitor of the 26S proteasome that is known to enter mitochondria where it can inhibit LONM^41^,^50^. Currently, the mechanism of action of MG132 on LONM is unclear, as is its effect on human CLPXP. To ensure that MG132 was indeed a specific inhibitor of human mitochondrial LONM (and not CLPXP) we monitored the degradation of α-casein *in vitro*, using either purified recombinant human LONM (Fig. 3A) or human CLPXP (Fig. 3B). Consistent with the findings of Orly and colleagues^41^ the LONM-mediated degradation of α-casein was inhibited by MG132 in a concentration dependent manner (Fig. 3A). Importantly, the CLPXP-mediated degradation of α-casein was not inhibited by MG132 even at concentrations (up to 400 μM) well in excess of those used to inhibit LONM. Having established that MG132 specifically inhibited LONM and not CLPXP activity, we applied these conditions to the mitochondrial import-chase proteolysis assay to determine if the turnover of OTC-Δ was mediated by LONM. Consistent with the effect of LONM-depletion on OTC-Δ stability, MG132 inhibited the turnover of OTC-Δ in a dose-dependent manner (Fig. 3C). Indeed at the highest concentration of MG132 used (50 μM), the turnover OTC-Δ was almost completely inhibited (Fig. 3C, filled squares) with ~80% of the protein remaining after a 2 h chase, in comparison to only ~20% remaining for the untreated mitochondria (Fig. 3C, open circles). Next, to confirm that LONM was directly responsible for the turnover of OTC-Δ we performed a series of immunoprecipitation (IP) experiments with LONM. Given that the interaction between a protease and its substrate is transient we performed the IP in the presence (and absence) of MG132 to inhibit LONM proteolytic activity and hence potentially trap OTC-Δ in the degradation chamber of the protease. Following import of OTC-∆ preprotein and incubation in the absence or presence of MG132, mitochondria were lysed. Endogenous LONM was then immunoprecipitated, using anti-LONM antiserum and compared to control IPs, using pre-immune serum. Importantly, OTC-∆ co-immunoprecipitated with LONM, but only in the presence of MG132 (Fig. 3D, lane 6). These data confirm that LONM interacts directly with OTC-∆ and is responsible for the degradation of this folding impaired protein in mitochondria.

### Unfolded but not aggregated proteins are degraded by LONM

**Figure 4:**
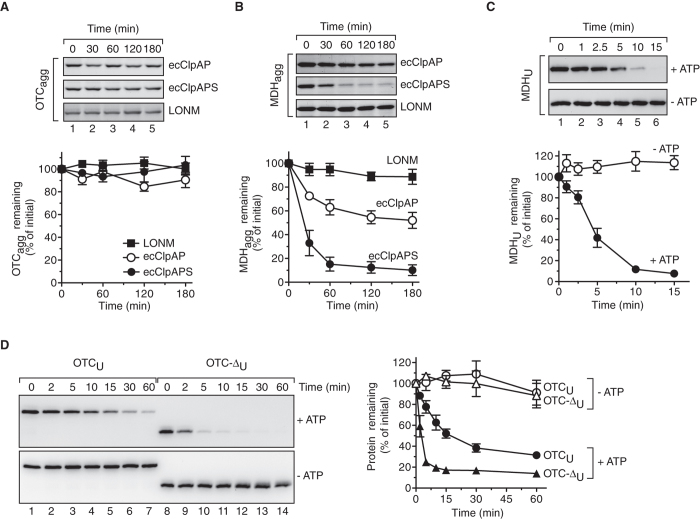
LONM recognises and degrades unfolded proteins. *In vitro* degradation assay monitoring the stability of aggregated OTC (**A**) or MDH (**B**) in the presence of *E. coli* ClpAP, ClpAPS or LONM as indicated. The relevant strips from Coomassie Brilliant Blue stained SDS-polyacrylamide gels are shown (upper panels). Quantification of three independent experiments is shown in the lower panels. Error bars represent SEM. *In vitro* degradation assay monitoring the stability of unfolded MDH (**C**), unfolded OTC (**D**) and unfolded OTC-∆ (**D**) in the presence of LONM with (filled symbols) or without (open symbols) ATP as indicated. Representative strips from anti-MDH (**C**), upper panel) and anti-OTC (**D**), left panel) immunoblots are shown. Quantification of at least three independent experiments respectively is shown for both unfolded MDH (**C**) and unfolded OTC/OTC-∆ (**D**). Error bars represent SEM.

Next we asked the question, which form of OTC-Δ (i.e. unfolded or aggregated) is recognised and degraded by LONM? To address this question, *in vitro* degradation assays were performed using either purified recombinant OTC or OTC-∆ together with a well-characterised model substrate, mitochondrial MDH^15^,^51^,^52^,^53^. Initially, we wanted to determine if LONM was capable of recognising and degrading aggregated OTC (OTC_agg_). To examine this, we first developed conditions to aggregate wild type OTC (Supplementary Fig. S1). Heat treatment of OTC at 55 °C but not at 47 °C or 50 °C, resulted in a rapid increase in turbidity (absorbance at 320 nm), reaching a maximum level within ~20 min (Supplementary Fig. S1A, filled circles). Following removal from the treatment, the aggregates were stable for >2 h at RT, with more than 95% of the heat-treated OTC remaining in the pellet fraction after centrifugation (Supplementary Fig. S1B, lane 6). Consistent with published data^52^, MDH was irreversibly aggregated following a 30 min incubation at 47 °C (Supplementary Fig. S1A, open circles and Supplementary Fig. S1B, lane 3). As such, in this study the thermal aggregation of OTC was performed at 55 °C for 30 min. Next, the stability of OTC_agg_ was monitored in the presence of either LONM (Fig. 4A, filled squares), *E. coli* ClpAP (Fig. 4A, open circles) or *E. coli* ClpAPS (Fig. 4A, filled circles). Neither ClpAPS nor LONM was able to degrade aggregated OTC. To validate these *in vitro* degradation assays, we used a well-established model aggregate, thermally aggregated MDH (MDH_agg_) as a control^51^,^52^,^54^. Importantly, and consistent with published data^51^, MDH_agg_ was efficiently degraded by *E. coli* ClpAP but only in the presence of ClpS (Fig. 4B, filled circles). However, degradation of MDH_agg_ was not observed in the presence of LONM (Fig. 4B, filled squares). Collectively, the data suggest that LONM is unable to degrade aggregated mitochondrial matrix proteins. These data also indicate that both aggregated proteins exhibit different properties, as ClpAPS was only able to degrade MDH_agg_ (Fig. 4B, filled circles) and not OTC_agg_ (Fig. 4A, filled circles).

Next, we monitored the *in vitro* degradation of unfolded MDH (MDH_U_) by LONM. In this case, MDH was chemically denatured (with guanidine HCl) using a well-established method^53^,^55^,^56^, then extensively diluted into a degradation buffer containing LONM. These data demonstrate that unfolded MDH (MDH_U_) was rapidly and completely degraded by LONM in an ATP-dependent manner (Fig. 4C, top panel), with a half-life of ~4 min, as determined from the quantification of four independent experiments (Fig. 4C, filled circles). As an additional control the stability of native MDH (MDH_N_) was also monitored in the presence of LONM (Supplementary Fig. S2A). Importantly, MDH_N_ was completely stable for the duration of the assay (180 min). Collectively, these data indicate that LONM specifically recognises and degrades MDH_U_ but not MDH_N_ or MDH_agg_. To determine if this behaviour was also true for folding impaired OTC-∆ responsible for the induction of UPR^mt^ in mammals, the stability of both unfolded OTC-∆ (OTC-∆_U_) and unfolded OTC (OTC_U_) was monitored in the presence of LONM (Fig. 4D). Purified recombinant mature OTC-∆ (isolated from inclusion bodies) and OTC (isolated from soluble lysate) were chemically denatured (as for MDH) and subsequently diluted into degradation buffer containing LONM. Importantly, in contrast to native OTC, which was stable in the presence of LONM and ATP (Supplementary Fig. S2B), both OTC_U_ and OTC-Δ_U_ were rapidly degraded by LONM in an ATP-dependent manner (Fig. 4D). Quantification of three independent degradation assays revealed that unfolded OTC-Δ (Fig. 4D, filled triangles) was degraded much more rapidly than unfolded OTC (Fig. 4D, filled circles) with a half-life of ~2 min compared to ~15 min for OTC_U_ (Fig. 4D). It is currently unclear why OTC-∆_U_ is degraded more rapidly than OTC_U_, but it is likely related to the nature of the degron presented to LONM in the unfolded state of the two proteins. Specifically, OTC-∆ is distinct from OTC, as it lacks 84 contiguous residues. Collectively these data demonstrate that LONM is responsible for the efficient recognition and degradation of unfolded proteins, but not of aggregated proteins.

Finally, as a control we monitored the turnover of unfolded OTC-∆ by purified recombinant human CLPXP (Supplementary Fig. S3). As expected and consistent with the import-chase proteolysis assays (Fig. 2) active CLPXP was unable to degrade OTC-∆_U_ (Supplementary Fig. S3A).

## Discussion

Maintenance of mitochondrial protein homeostasis and bioenergetics is fundamental to health and normal aging of eukaryotic organisms^14^. It is achieved through multiple layers of interrelated processes, from chaperone- and protease-mediated PQC, to activation of the mitochondrial unfolded protein response and finally the selective destruction of mitochondria by mitophagy^14^. In mammalian cell lines, the overexpression of a folding-impaired mitochondrial protein OTC-∆ has been shown to induce both UPR^mt^ and mitophagy in energised mitochondria^25^,^26^. Currently however, the trigger for activation of these processes in mammals is unknown. Nevertheless, several studies in *C. elegans* have revealed that the mitochondrial matrix protease ClpXP contributes to the UPR^mt^ signalling pathway and it has been proposed that peptides (or cofactors), derived from CLPXP-mediated degradation of unfolded proteins act to transmit the stress signal^30^,^31^. Here we have tracked the fate of the UPR^mt^ inducing protein OTC-∆ in human mitochondria to gain insight into the contribution that the matrix proteases (CLPXP and LONM) make to protein homeostasis in human mitochondria.

The apparent turnover of mitochondrial proteins can be attributed to either the selective degradation of the protein by resident proteases or the selective degradation of mitochondria by the lysosome^57^,^58^,^59^,^60^. By examining the fate of OTC-∆ imported into isolated mitochondria (Fig. 1) rather than in cells we have established that this aggregation-prone protein is unstable and selectively cleared from within the organelle in an ATP-dependent manner. Given that the ClpXP protease was shown to play an important role in the UPR^mt^ signalling pathway in *C. elegans* we speculated that human CLPXP would be responsible for the degradation of OTC-∆. Surprisingly, and contrary to the proposed model of UPR^mt^ signal transduction in humans, our data demonstrated that the rate of OTC-Δ degradation in both CLPX- and CLPP-depleted mitochondria was unchanged relative to control mitochondria (Fig. 2), indicating that CLPXP does not contribute to the removal of OTC-Δ in human mitochondria. In contrast, the loss of LONM function in mitochondria, either through depletion of LONM by RNAi-mediated knock-down or inactivation of LONM by MG132, almost completely stabilised OTC-Δ with ~80–85% of the protein remaining after a 2 h incubation at 37 °C (Figs 2 and 3). Collectively these data indicate that the turnover of OTC-Δ in mitochondria is largely mediated by LONM. We cannot however exclude the possibility that the *m*-AAA proteases or other unknown ATP-dependent proteases play a minor role in the turnover of OTC-∆.

The apparent inability of CLPXP to degrade OTC-∆ suggests that human CLPXP may not degrade folding intermediates of mitochondrial matrix proteins. These data also suggest that, UPR^mt^ signalling in human mitochondria may not be linked to the CLPXP-mediated degradation of unfolded proteins. It could however be argued that CLPXP is responsible for the turnover of a specific mitochondrial matrix protein (or proteins) that only becomes accesible when the PQC machinery is over-burdened. However, it was recently revealed that overexpression of OTC-∆ in a human cell line induces mitophagy in polarised mitochondria via a PINK1 (PTEN-induced putative kinase 1) and PARK2 (Parkin E3 ubiquitin ligase) dependent pathway^26^. Consistent with our identification of LONM as the major protease responsible for the turnover of OTC-∆, Youle and colleagues revealed that depletion of LONM (and not CLPP) enhanced PINK1 and PARK2 recruitment (and hence mitophagy) to mitochondria exposed to unfolded protein stress. Hence CLPP does not modulate mitophagy because it does not contribute to the clearance of misfolded proteins in mitochondria, while in contrast LONM, as the central PQC protease, is a key moderator of mitophagy when matrix protein homeostasis in compromised. Interestingly, mutations in *CLPP* are associated with Perrault syndrome 3 (MIM 614129) which is characterised by sensorineural hearing loss and premature ovarian failure^19^ and *CLPP* knockout mice display phenotypes consistent with Perrault syndrome as well as other distinct effects such as elevated mtDNA levels and induction of selected matrix protein quality control components and inflammatory factors^61^. Hence we speculate that human CLPXP may contribute to the temporal degradation of specific proteins for the purpose of regulating mitochondrial homeostasis or cellular processes, rather than contributing to the direct and promiscous degradation of unfolded or misfolded proteins for general PQC. However, this model requires further examination through the direct determination and comparison of the human CLPXP substrate proteome under normal and protein folding stress conditions.

In contrast to CLPXP, several substrates of human LONM have been defined. Human LONM is responsible for the turnover of mildly oxidised proteins such as aconitase as well as unassembled proteins^38^,^39^,^40^,^62^. LONM is also responsible for the regulated turnover of natively folded mitochondrial proteins including mammalian steroidogenic acute regulatory protein (StAR), 5-aminolevulinic acid synthase 1 (ALAS-1) and mitochondrial transcription factor A (TFAM)^41^,^42^,^43^,^62^. To further dissect the role of AAA+ proteases in human mitochondrial homeostasis we examined the specificity of LONM using three mitochondrial matrix proteins MDH, OTC and OTC-Δ in different conformational states (native, unfolded and aggregated). Strikingly, when all three proteins were chemically unfolded and diluted into aqueous solution containing LONM, they were rapidly degraded in an ATP-dependent manner. This is consistent with *E. coli* Lon (ecLon), which recognises short hydrophobic polypeptide stretches on proteins that are usually buried in the folded protein and exposed on the surface only upon unfolding^7^,^63^. Interestingly, the rate of degradation of unfolded OTC-Δ (t ½ ~ 4 min) was much faster than that of unfolded OTC (t ½ ~ 15 min) (Fig. 4). We speculate that this is due to the exposure of different hydrophobic residues or patches in the unfolded state of these proteins, which may be recognised with different affinities. Alternatively, the faster rate of turnover (of OTC-Δ) may result from reduced folding kinetics of the mutant protein facilitating increased partitioning of the substrate to the protease. Consistently, ecLon exhibited different degradation rates for truncated products of β-galactosidase^63^,^64^. Here we provide direct evidence that LONM has the capacity to bind and degrade unfolded or misfolded states of proteins, likely due to exposure of hydrophobic residues that are normally buried in the native protein.

Since the *E. coli* AAA+ protease ClpAPS is known to recognise and degrade aggregated proteins^51^ we questioned whether a human AAA+ protease also exhibited such an activity. To examine this, we monitored the stability of MDH_agg_ and OTC_agg_ *in vitro* in the presence of LONM (Fig. 4). Over a 3-hour incubation, in the presence of ATP regenerating system, little to no degradation of either aggregated MDH or OTC was observed. This data suggests that aggregated mitochondrial matrix proteins are either degraded very slowly by LONM or are not substrates of this protease. This is consistent with a previous study showing aggregated aconitase (formed due to severe oxidative damage) is a poor substrate of LONM^39^. Similarly, LONM was unable to degrade insoluble yeast MPPα generated by either chemical or heat denaturation^62^. Thus, LONM appears to recognise and degrade the soluble unfolded or misfolded state of proteins before they aggregate. Consistently, Lon-deficient (Δ*pim1*) yeast cells show an accumulation of aggregated mitochondrial polypeptides following treatment with heat, hydrogen peroxide (H_2_O_2_) or menadione^65^, while electron microscopy images of mitochondria isolated from either Δ*pim1* yeast cells or LONM-depleted human lung fibroblasts (WI-38 VA-13) revealed mitochondria with electron-dense inclusions within the organelle, which likely represent protein aggregates^66^,^67^. Furthermore, cells derived from patients with CODAS syndrome, a multisystem developmental disorder caused by mutations in *LONP1* (coding for LONM), contain electron dense inclusions within mitochondria^68^.

Despite the presence of a CLPXP protease in human mitochondria, our data suggests that LONM is the central PQC protease in the mammalian mitochondrial matrix. Interestingly, the unfolded states of the substrates examined in this study (MDH and OTC-∆) also interact with human HSP60 both *in vitro* and *in organello*^15^,^25^. Therefore unfolded proteins are likely to partition between LONM and molecular chaperones such as HSP60. Consequently, any factor that slows the kinetics of protein folding (e.g. genetically inherited mutation), is also likely to reduce its half-life due to increased degradation by LONM. Since HSP60, and not LONM, is up-regulated by UPR^mt^ 35 it seems important for mitochondria (under condition of protein folding stress) to prevent aggregation and recover folded functional proteins rather than removing them by proteolysis. In the case that the folding and degradation capacity of the mitochondrial matrix is exceeded, aggregates will accumulate. In bacteria and eukaryotes such as yeast, mechanisms exist in the cell to recover natively folded functional proteins from misfolded amorphous aggregates. This process is mediated by the AAA+ protein ClpB (Hsp78 in yeast mitochondria) together with DnaK (Ssc1p in yeast) and cofactors^52^,^69^,^70^. To date a functionally equivalent machinery has not been described in human mitochondria. Thus, in the absence of a mechanism to clear aggregates from within mitochondria (by degradation or dissaggregation and refolding) it seems the selective clearance of compromised mitochondria by mitophagy may play a critical role in maintaining mitochondrial protein homeostasis.

## Methods

### *In vitro* transcription and translation

*In vitro* transcription of *Rattus norvegicus* OTC precursor was performed using pSP65/*pre-OTC*^71^. To generate a plasmid for *in vitro* transcription of *OTC-*Δ, pSP65/*pre-OTC* was digested with *Bgl* II^25^, releasing a 255 bp insert and the linear plasmid ligated to generate pSP65*/pre-OTC-*Δ. To generate radiolabelled pre-OTC and pre-OTC-∆, *in vitro* transcription and translation was performed. Briefly, pSP65*/pre-OTC* and pSP65*/pre-OTC-*Δ were digested with *Sma* I then used as template to synthesise mRNA using SP6 RNA polymerase (Promega). The ^35^S-labelled precursor proteins were synthesised from *pre-OTC* and *pre-OTC-∆* mRNA in rabbit reticulocyte lysate (Promega), supplemented with 11 μCi [^35^S]Met/Cys EXPRE^35^S^35^S protein labelling mix (specific activity >1000 Ci/mmol; Perkin Elmer).

### Expression and purification of proteins

For the bacterial expression of mature OTC and OTC-Δ, the appropriate cDNAs were amplified by PCR (using Nde_OTC 5′- CTGATCCATATGAGTCAAGTACAGCTGAAAGGCCG-3′ and ratOTC_Not 5′- GATGATGCGGCCGCGAACTTTGGCTTCTGGAGCAC-3′), digested with *Nde* I and *Not* I, then ligated into a similarly digested plasmid, pET10C^72^ to generate pET10C*/OTC* and pET10C*/OTC-*Δ, respectively. His_10_-tagged mature recombinant OTC and OTC-∆ were expressed at 20 °C, in BL21-CodonPlus (DE3)-RIL (Agilent Technologies) *E. coli* cells bearing pET10C*/OTC* and pET10C*/OTC-*Δ, respectively. Following expression, cells were lysed in 50 mM Tris-HCl pH 8.0, 300 mM NaCl, 1 mM EDTA, 0.05% (v/v) Triton X-100, 10% (v/v) glycerol, 0.2 mg/ml egg white lysozyme, 10 μg/ml DNase I and 5 mM MgCl_2_, and mature recombinant OTC was isolated from the clarified lysate via Ni-NTA agarose affinity chromatography (Qiagen). The soluble lysate was applied to Ni-NTA agarose beads in equilibration buffer (50 mM Tris-HCl pH 8.0, 300 mM NaCl) supplemented with 10 mM imidazole. Beads were washed step-wise using equilibration buffer containing 10 mM, 20 mM, 65 mM and finally 100 mM imidazole (5 bed volume per step). Mature recombinant OTC was eluted using equilibration buffer containing 500 mM imidazole. OTC-Δ was also purified from inclusion bodies using a method adapted from Schulz and colleagues^73^. Cells were resuspended in 50 mM Tris-HCl pH 8.0, 100 mM NaCl containing 0.1 mg/ml egg white lysozyme, 0.5 mM PMSF, EDTA free protease inhibitor cocktail (Roche) at a ratio of 5 ml buffer/1 g cells (wet weight) and incubated for 30 min at RT. Next, the suspension was incubated (30 min at RT) with sodium deoxycholic acid (4 mg/g wet cells) and DNase I (10 μg/ml) before the crude inclusion body pellet was collected by centrifugation (3019 *g* for 60 min at 4 °C). Finally the inclusion body fraction was then washed sequentially by resuspension (with stirring) in 50 mM Tris-HCl pH 8.0, 1 mM EDTA, 100 mM NaCl, 10 mM DTT, 2% (v/v) Triton X-100 (30 min at 4 °C), then 50 mM Tris-HCl pH 8.0, 1 mM EDTA, 100 mM NaCl, 10 mM DTT (2 h at 4 °C) and 50 mM Tris-HCl pH 8.0, 100 mM NaCl (30 min at 4 °C). Between each wash, the inclusion bodies were collected by centrifugation (3019 *g*, 30 min, 4 °C). Recombinant mature human LONM was expressed in *E. coli* from pET10C/*LONP1* and purified as described previously^38^. Recombinant mature human CLPX and CLPP were expressed in *E. coli* from pET10C/*hCLPX* and pHUE/*hCLPP* and purified as described previously^37^,^38^. Purified recombinant *E. coli* ClpA, ClpP and ClpS were prepared as described previously^51^. Pig heart mitochondrial malate dehydrogenase (MDH) and α-casein were from Sigma-Aldrich.

### Cell culturing, mitochondrial isolation and protein import

HeLa cells were grown at 37 °C in the presence of 5% (v/v) CO_2_ in Dulbecco’s Modified Eagle Medium (Life Technologies) supplemented with 10% (v/v) fetal calf serum (Life Technologies). Transfection of cells with 20 nM siRNA (Life Technologies) was performed using Lipofectamine 2000 (Life Technologies). Silencer or Silencer select siRNA directed against *CLPX* (s21289) *LONP1* (s17903) and *CLPP* (105684) and the appropriate negative control siRNA, were used. Isolation of mitochondria and *in vitro* import of radiolabelled protein was performed essentially as described previously^74^,^75^.

### Preparation of unfolded proteins

For protease mediated degradation assays, soluble unfolded proteins were prepared using guanidine hydrochloride. Specifically, either native MDH (8.6 μM), native OTC (4.3 μM) or 4.3 μM of OTC-Δ (isolated inclusion bodies) were incubated in 48.7 mM Tris-HCl pH 7.8, 6.9 M guanidine hydrochloride and 1.98 mM DTT at 0 °C for 30–60 min essentially as described previously for MDH unfolding^53^. For degradation assays the unfolded proteins were diluted 50-fold (final protomer concentration of MDH and OTC were 0.172 μM and 0.086 μM respectively) into proteolysis buffer containing 1.2 μM LONM (protomer) or where relevant 2.4 μM CLPX (protomer) and 5.6 μM CLPP (protomer).

### Thermal aggregation of proteins

Thermal aggregation of natively folded OTC or MDH was performed as previously described for MDH^51^,^52^. Briefly, 2 μM of substrate protein was incubated in aggregation buffer (100 mM Tris-HCl pH 7.5, 150 mM KCl, 20 mM MgCl_2_, 10 mM DTT) for 0–60 min at either 0 °C, 47 °C, 50 °C or 55 °C. Aggregate formation (turbidity) was determined by measuring the change in absorbance at 320 nm using 10 mm quartz cuvette and a SpectraMax M5 spectrophotometer (Molecular Devices). For fractionation of thermally aggregated proteins, following incubation of either MDH (2 μM) or OTC (2 μM) in aggregation buffer for 30 min at either 0 °C, 47 °C, 50 °C or 55 °C, the soluble and insoluble fractions were separated by centrifugation (at 16 060 *g*) at room temperature for 30 min. For degradation assays, solutions of aggregated MDH (aggregated at 47 °C for 30 min) or aggregated OTC (aggregated at 55 °C for 30 min) were added to proteolysis buffer containing 1.8 μM LONM (protomer).

### Protein degradation assays

Turnover of substrates by LONM protease was performed essentially as described previously^36^. In brief, native, unfolded or aggregated protein substrates (86–3000 nM) were incubated together with LONM (0.6–1.8 μM, protomer) in proteolysis buffer (50 mM Tris-HCl pH 8.0, 100 mM KCl, 20 mM MgCl_2_, 0.02% (v/v) Triton X-100, 10% (v/v) glycerol, 1 mM DTT) at 30 °C for 5 min with degradation initiated by the addition of ATP (5 mM). The degradation of aggregated substrates by *E. coli* ClpA (1.2 μM), ClpP (2.8 μM) and ClpS (1.2 μM) was performed in proteolysis buffer, essentially as described^51^. Turnover of α-casein (3 μM) or unfolded OTC-Δ (0.086 μM) by human CLPX (2.4 μM) and human CLPP (5.6 μM) was performed in the presence of 5 mM ATP but without ATP regeneration as described^37^. Where indicated, MG132 (5 μM–400 μM) in ethanol or ethanol alone (control), was added to the proteolysis mix. Unless otherwise stated degradation assays longer than 90 min, were also supplemented with an ATP-regeneration mix (4 μg/ml pyruvate kinase, 4 mM phosphoenolpyruvate). To terminate reactions, samples were mixed with SDS-PAGE loading buffer followed by heat treatment at 95 °C. To monitor protein degradation within isolated mitochondria, import-chase proteolysis assays were performed^38^,^44^. Radiolabelled precursor protein (OTC or OTC-Δ) were imported into energised isolated mitochondria for 7.5 min at 37 °C. Following dissipation of the membrane potential with valinomycin (2 μM), non-imported precursor protein was removed by trypsin treatment (5 μg/ml) followed by inhibition of trypsin with soybean trypsin inhibitor (1 mg/ml). Prior to the chase (incubation for 120 min), mitochondria were either left untreated or treated with ATP regeneration mix (5 mM ATP, 10 mM creatine phosphate, 100 μg/ml creatine kinase), ATP depletion mix (40 U/ml apyrase, 20 μM oligomycin), MG132 (10 or 50 μM) in ethanol or ethanol alone (control). For the immunoprecipitation experiments radiolabelled OTC-Δ preprotein was freshly imported in isolated mitochondria, then following trypsin treatment supplemented with either MG132 (50 μM) in ethanol or ethanol alone (control) and incubated at 37 °C for 5 min. Mitochondria were reisolated, washed, then resuspended in IP buffer (50 mM Tris-HCl pH 7.5, 100 mM KCl, 10 mM Mg-acetate, 5% (v/v) glycerol, 0.5% (v/v) Triton X-100) and incubated on ice for 30 min, with occasional mixing. Soluble mitochondrial lysate was applied to antibodies (from either anti-LONM or pre-immune serum) cross-linked to Protein A Sepharose and incubated with gentle mixing for 1 h at 4 °C. Following wash steps, antibody-bound proteins were eluted using 50 mM glycine-HCl pH 2.5 followed by neutralisation of the sample.

### Detection of proteins

Following separation by glycine- or Tricine-buffered SDS-PAGE^76^, proteins were detected either by Coomassie Brilliant Blue R250 staining, digital autoradiography or immunoblotting. Dried radioactive gels were exposed to phosphor screens and scanned using the Typhoon^TM^ Trio variable mode imager (GE Healthcare). Analysis and quantification of gel images was performed using the ImageQuant software (Version 5.1, GE Healthcare). For the analysis of experiments by both digital autoradiography and immunoblotting, proteins were first transferred from polyacrylamide gels to polyvinylidene difluoride (PVDF) membrane using a semi-dry transfer method, before the dried membrane was exposed to a phosphor screen for analysis. The same membrane was then analysed by immunoblotting with the appropriate antisera (directed against human LONM, CLPX, CLPP and NDUFA9) as described previously^38^. Antiserum against rat OTC, was kindly provided by Nick Hoogenraad (La Trobe University). MDH antibody-horse radish peroxidase (HRP) conjugate (GTX40570) was from GeneTex. In general, membrane bound primary antibodies were detected using HRP-coupled secondary antibodies (anti-mouse or anti-rabbit IgG, Sigma-Aldrich) incubated with enhanced chemiluminescence detection reagents (GE Healthcare) and images captured with a ChemiDoc XRS+ system (Bio-Rad) using Image Lab Software (Bio-Rad).

## Additional Information

**How to cite this article**: Bezawork-Geleta, A. *et al.* LON is the master protease that protects against protein aggregation in human mitochondria through direct degradation of misfolded proteins. *Sci. Rep.* **5**, 17397; doi: 10.1038/srep17397 (2015).

## Supplementary Material

Supplementary Information
