# Supplementary material for: LON is the master protease that protects against protein aggregation in human mitochondria through direct degradation of misfolded proteins
Source: Sci Rep. 2015 Dec 2;5:17397. doi: 10.1038/srep17397 (PMC4667172; doi:10.1038/srep17397)
Supplement: Supplementary Information [file srep17397-s1.pdf]

LON is the master protease that protects against protein aggregation in human mitochondria through direct degradation of misfolded proteins

Ayenachew Bezawork-Geleta[1,2], Erica J. Brodie[1], David A. Dougan[1*] and Kaye N. Truscott[1*]

1. Department of Biochemistry and Genetics, La Trobe Institute for Molecular Science, La Trobe University, Melbourne 3086, Australia.

2. Present address: School of Medical Science, Griffith University, Southport, QLD 4222, Australia.

*, Correspondence: Kaye N. Truscott, Tel: +61 3 9479 5245; Fax: +61 3 9479 1266; E-mail: k.truscott@latrobe.edu.au or David A. Dougan, Tel: +61 3 9479 3276; Fax: +61 3 9479 1266; E-mail: d.dougan@latrobe.edu.au.

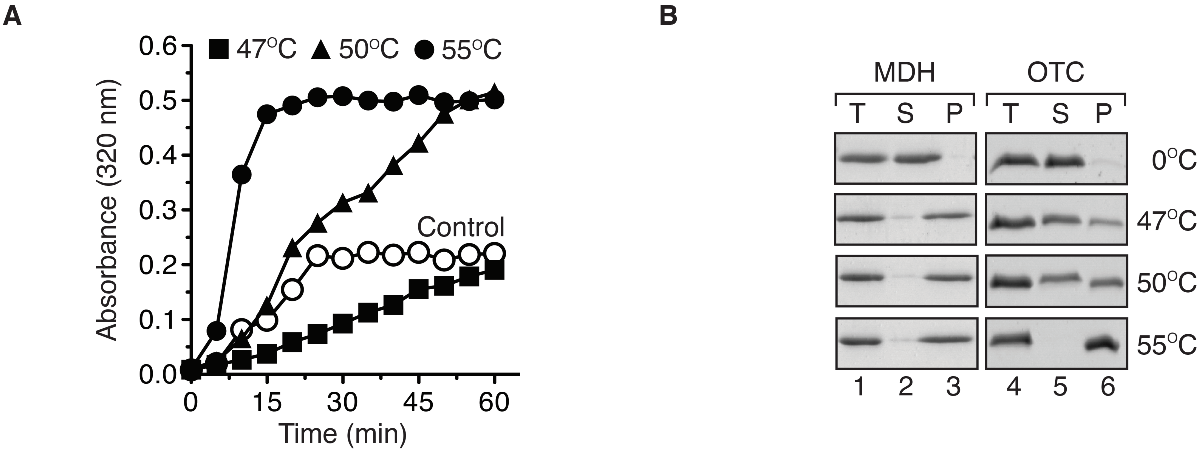

Supplementary Figure S1: Thermal denaturation of OTC

A) Thermal denaturation of OTC (filled symbols) at the indicated temperatures monitored by light scattering (320 nm) alongside the MDH control treated at 47°C (open symbols). B) Thermal denaturation of OTC and MDH (control) at the indicated temperatures monitored by fractionation. Samples were separated by SDS-PAGE and stained with Coomassie Brilliant Blue. T, total protein input; S, soluble fraction following centrifugation, P, pellet fraction following centrifugation.

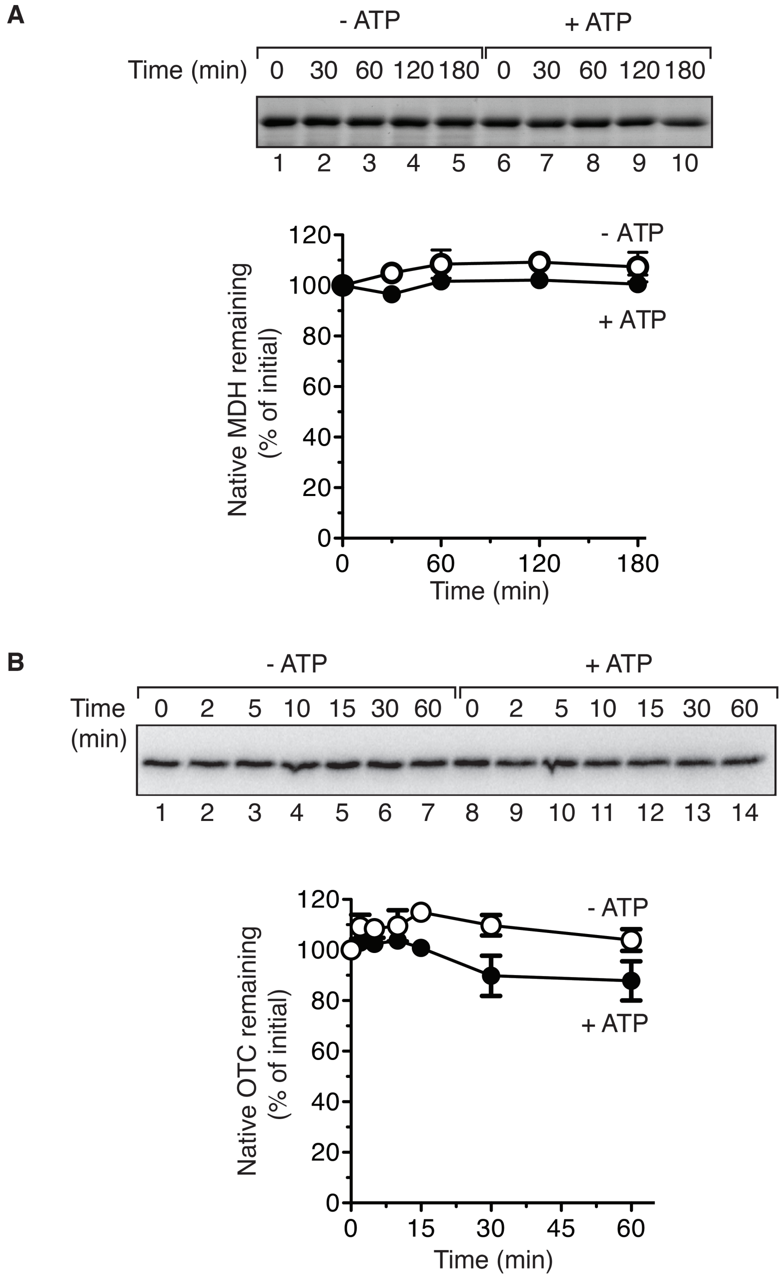

Supplementary Figure S2: Native MDH and OTC are not substrates of LONM

In vitro degradation assay monitoring the stability of natively folded MDH (A) or OTC (B) in the presence of LONM with and without ATP as indicated. The relevant strips from Coomassie Brilliant Blue stained SDS-polyacrylamide gels are shown (upper panels). Quantification of four independent experiments is shown in the lower panels. Error bars represent SEM.

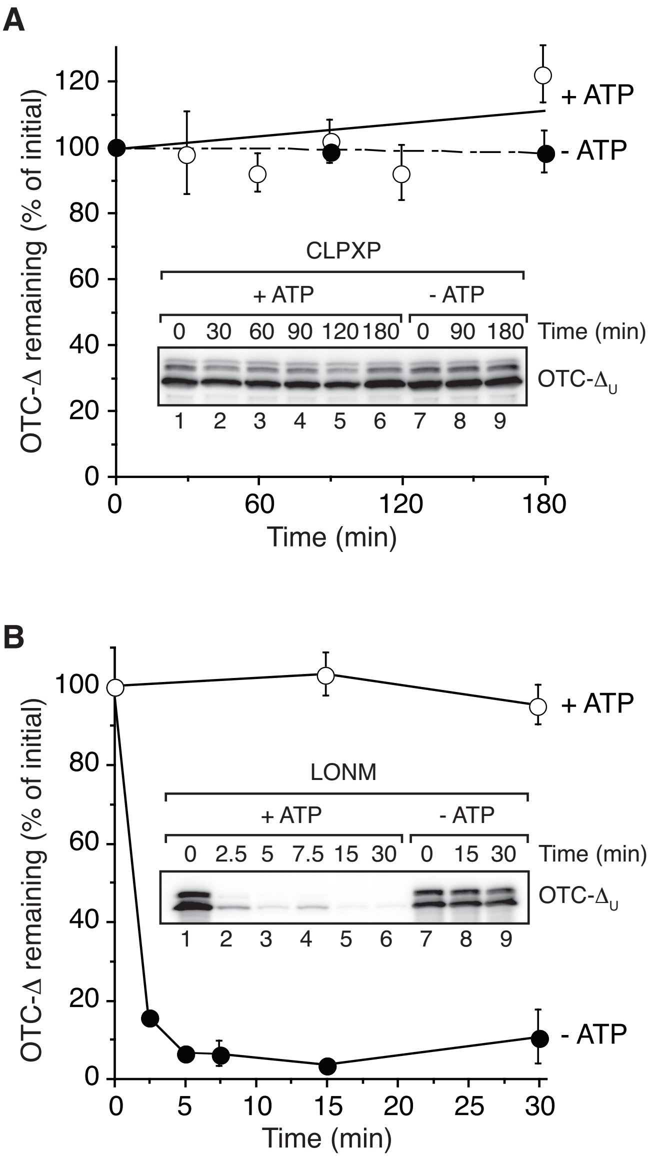

Supplementary Figure S3: Unfolded OTC-Δ is not a substrate of human CLPXP. Quantitations showing the *in vitro* stability of urea denatured recombinant OTC-Δ in the presence of active CLPXP (A) or LONM (B) with or without ATP as indicated. Error bars represent SEM for three (A) or two (B) independent experiments. Representative strips from immunoblots (anti-OTC) are shown (inset).
